# Effects of supplementing n-3 fatty acid enriched eggs and walnuts on cardiovascular disease risk markers in healthy free-living lacto-ovo-vegetarians: a randomized, crossover, free-living intervention study

**DOI:** 10.1186/1475-2891-13-29

**Published:** 2014-03-27

**Authors:** Bonny Burns-Whitmore, Ella Haddad, Joan Sabaté, Sujatha Rajaram

**Affiliations:** 1 Department of Nutrition, School of Public Health, Loma Linda University, Loma Linda, CA 92350, USA; 2 Department of Human Nutrition and Food Science, California State Polytechnic University, Pomona, 3801 West Temple Ave, Pomona, CA 91768, USA

**Keywords:** Walnuts, n-3 fatty acid enriched eggs, Lacto-ovo vegetarians, Cardiovascular risk factors, Eggs, Inflammation markers, Blood lipids

## Abstract

**Background:**

Plant and marine n-3 fatty acids (FA) may favorably modify select markers of cardiovascular disease risk. Whether supplementing the habitual diet of lacto-ovo-vegetarians (LOV) with walnuts (containing α-linolenic acid, ALA) and n-3 FA enriched eggs (containing primarily docosahexaenoic acid, DHA and ALA) would have equivalent effects on CVD risk factors is explored in this study.

**Methods:**

In this study, 20 healthy free-living LOVs following their habitual diet were randomly assigned in a crossover design to receive one of three supplements: n-3 FA enriched egg (6/week), walnuts (28.4 g, 6/week) or a standard egg, 6/week (control) for 8 weeks each with 4-wk washout between treatments. Erythrocyte membrane fatty acids, serum lipids and inflammatory markers were measured at the end of each treatment.

**Results:**

Dietary compliance was observed by an expected increase in erythrocyte membrane ALA following the walnut treatment and in DHA following the n-3 FA enriched egg treatment. Walnut treatment lowered serum triacylglycerol, total cholesterol and Apo B (p < 0.05) compared to the standard egg but not the n-3 FA enriched egg treatment. However, walnut treatment significantly reduced total: HDL cholesterol ratio compared to both egg treatments. There were no differences between treatments for any of the inflammatory markers.

**Conclusions:**

For LOV, a direct source of DHA such as n-3 FA enriched eggs seems necessary to increase membrane levels of DHA. However for producing an overall favorable blood lipid profile, daily consumption of a handful of walnuts rich in ALA may be a preferred option for lacto-ovo vegetarian.

## Background

The major plant n-3 fatty acid α-linolenic acid (ALA) is found in walnuts, soybean, canola oil, and flaxseed; while the major marine n-3 fatty acids, eicosapentaenoic (EPA) and docosahexaenoic acids (DHA), are found in cold-water fatty fish and flax or soy-fed poultry and farm animals [1,2]. Diets containing high amounts of both plant and marine derived n-3 fatty acids are associated with reduced risk of cardiovascular disease (CVD) [3,4], but studies indicate Americans do not consume enough n-3 fatty acids [1,5,6]. The cardio-protective effects of n-3 fatty acids may be partially mediated via favorable changes in blood lipids and inflammation [7,8]. Whether or not ALA and EPA/DHA have equivalent effects on CVD risk factors specifically in healthy lacto-ovo-vegetarians (LOV) has not extensively studied.

Some fortified dairy products and eggs contain very small amounts of DHA (0.02-0.3 g/serving) and are the only direct sources of DHA in the LOV diet [1,6]. The only vegan DHA option is an algae-based supplement [9]. Vegans and LOVs depend on the endogenous production of EPA and DHA from ALA; however, this endogenous conversion is inefficient with only 5-7% of dietary ALA converting to EPA and DHA [10]. The dietary ratio of n-6 to n-3 fatty acids influences conversion of ALA to EPA and DHA [6]. While LOVs and vegans do consume significant amount of ALA in their diet, overall their linoleic acid (LA) intake is many folds higher than ALA intake producing high n-6: n-3 fatty acid ratio. This may suppress the conversion of ALA to EPA and DHA (1, 6).

Consumption of 57-71 g/day of ALA rich walnuts decreases total and LDL cholesterol (LDL-C) and triacylglycerol (TAG) in normo- and hypercholesterolemic individuals, resulting in a favorable reduction in CVD risk (11-16). Studies with cold-water fatty fish demonstrate reduction of atherosclerotic plaque formation, lowering of TAG and an increase in HDL-C, supporting the rationale for including fatty fish in the diet to prevent Coronary Heart Disease [3,11-13]. We recently examined the effects of consuming fatty fish and walnuts on serum lipids and found that walnuts lowered total and LDL-C while the fatty fish decreased serum TAG and increased HDL-C [14]. Since LOVs do not consume fish, an alternate source of long chain n-3 fatty acids for LOVs could be n-3 fatty acid enriched eggs. Chickens fed flaxseed, soy, or algal marine sources, incorporate DHA and ALA into their eggs, which results in the production of n-3 fatty acid enriched eggs [15,16].

N-3 fatty acid enriched eggs fed to hypercholesterolemic non-vegetarians showed increase in HDL-C, decrease in platelet aggregation, and significant lowering of TAG [17-20]. Some studies have looked at the effect of feeding n-3 fatty acid enriched eggs on blood lipids, but none have compared the effects of ALA-rich walnuts with that of n-3 fatty acid (primarily DHA) enriched egg on CVD risk factors especially among LOVs.

## Methods

### Participants

Healthy LOV subjects were recruited from southern California via a multistage screening process with community advertisements through local newspapers and flyers. Potential candidates that responded were presented with the study information at group meetings and were asked to sign the informed consent if they decided to proceed with the study. Screening process also included interviews with two investigators to assess potential compliance. Men and women between the ages of 21-90 years and who had normal total cholesterol (Total-C) and TAG levels were included in the study. Participants were excluded if they had existing medical conditions, were <21 or ≥ 90 years of age, were smokers, drank alcohol, took supplements, not willing to follow dietary intervention and instructions, were on cholesterol lowering medications, or on daily prescription anti-inflammatory medication, and were LOV for < 3 months. The minimum sample size required for a crossover design to detect a mean absolute difference in serum LDL-C of 0.26 mmol/L at α =0.05 and power > 80% was calculated to be 18. Assuming a dropout rate of 20%, we recruited 26 subjects for this study. All participants signed a written informed consent approved by the Internal Review Boards at Loma Linda University, Loma Linda, CA and California State Polytechnic University, Pomona, CA.

### Study design and protocol

The study was a randomized, crossover (3 × 3 Latin-square), free-living intervention study in which subjects were blinded to the egg treatments. Participants were asked to follow their habitual diet and add the treatment food as a supplement. They were randomly assigned to n-3 fatty acid enriched egg (6/wk), walnuts (28.4 g, 6x/wk) or standard egg, 6/wk (control). Treatments were followed for 8 weeks each in a crossover design, with a 4-week washout period between treatments. Previous studies [14,21,22] have shown that 3-6 weeks intervention and 4 weeks washout is adequate to see changes especially for serum lipids and red cell membrane fatty acids.

The n-3 fatty acid enriched eggs were obtained from chickens fed an organic, vegetarian diet containing flaxseed and the standard eggs for the control treatment were obtained from chickens fed organic, vegetarian diet. The n-3 fatty acid composition of the two eggs was previously published [23]. The n-3 fatty acid enriched egg contained ~500 mg DHA, 40 mg EPA and 1 g ALA per egg yolk while the standard egg yolk contained 110 mg DHA, negligible amount of EPA and 0.15 g ALA. The walnuts used in the study provided 2.95 g ALA and 10.4 LA per 28.4 grams or 1 ounce of walnuts (Covance, Madison, WI).

Participants received diet counseling and their intervention foods from a Registered Dietitian (RD) every two weeks during the treatment periods. Participants were asked to avoid excess amounts of foods rich in n-3 fatty acids such as flax, soy, or canola oil, and to refrain from consuming any additional eggs or walnuts other than the ones supplied during the study. The instructions provided to the subjects were to add the treatment food to their habitual diet. They could incorporate the foods in any form they desired. Eggs could be cooked as omelet or hard boiled eggs or incorporated into a dish provided the participant consumed the entire serving. Walnuts could be eaten raw or used on salads, muffins, etc. Diet and caloric consumption was determined by doing 24-hour recalls. This was a free-living study, so there were no instructions provided regarding caloric intake. Subjects were asked to maintain the same lifestyle habits with which they entered the study such as the level and type of physical activity.

### Quality control

Quality control and compliance was ensured among study participants by: 1) counseling with the RD throughout the study; 2) maintaining a daily diary to record deviations in food and medicine intake; 3) obtaining three 24-hour recalls during each treatment period to assess nutrient intake (Nutrition Data System-Research software -NDS-R, Nutrition Coordinating Center, Division of Epidemiology © 2007 by the Regents of the University of Minnesota); 4) assessing fatty acid composition of erythrocyte membranes at the end of each treatment period to assess compliance to treatment food (Lipomics Technologies, West Sacramento, CA).

### Data collection

Participant’s body weight was assessed at baseline and end of all treatments (Tanita TBF 310-GS scale, Tanita Corporation of America Inc., Arlington Heights, IL, USA). Twelve-hour fasting blood samples were collected at baseline and end of each treatment period. Serum was separated by centrifugation, aliquoted and stored at -80°C until further analyses. The erythrocytes were washed and stored in saline at –80°C until further analyses.

### Analyses of serum markers

Lipomics Technologies (West Sacramento, CA) extracted and analyzed the erythrocyte membrane lipids as described previously [24]. The Nutritional Assessment Core at the University of California, Davis (supported by NIH NIDDK grant 35747), performed the serum lipid and lipoprotein analyses [14,24]. Serum interleukin 1β (IL-1β), interleukin 6 (IL-6), tumor necrosis factor alpha (TNF-α), soluble intracellular adhesion molecule-1 (sICAM-1), and sE-Selectin were analyzed utilizing the quantitative high sensitivity technique according to the supplier’s instructions (R&D Systems, Minneapolis, MN). High-sensitivity C-reactive protein (Hs-CRP) was analyzed using Enzyme-linked immunosorbent assay (Diagnostic Systems Laboratories, Inc., Webster, TX).

### Statistical analysis

Analysis was carried out using the SAS Mixed Procedure (SAS/STAT software, Version 9.2 of the SAS/SAT System for Unix, 2008. SAS Institute Inc., Cary, NC), which correctly analyzes data with heterogeneous variances. A heterogeneity model was used for arachidonic acid and total n-3 fatty acids, and homogeneity model was used for all other nutrients. When ANOVA was significant (P < 0.05), paired comparison tests among the treatments were carried out, and adjusted for multiple comparisons by using the Tukey-Cramer method. Results are presented as least-squares means and 95% CIs after back-transformation.

Erythrocyte membrane fatty acids, blood lipids and inflammatory factors data were analyzed by using mixed linear models that included fixed terms for treatment and period, and a random term for subjects. TAG, HDL-C and all inflammatory factors except sICAM, were normalized by log-transformation. Results for these outcomes were transformed back into their original units, and are presented as least-squares geometric means and their 95% C.I. Results with a P value of <0.05 were considered statistically significant.

Dietary EPA and DHA, which contained many zero-valued observations, could not be normalized by transformation. Therefore, tests for significant differences among treatments were carried out by using two-way, fixed-effects ANOVA on the ranks of the subject means. When ANOVA was significant, paired comparison tests were used, and adjusted for multiple comparisons.

Baseline values for the variables are not shown in tables as this is a crossover study design where the comparison between each treatment is more relevant than comparison to baseline; however, the baseline-adjusted analyses was carried out utilizing fixed covariates representing the period baseline and the subject-average period baselines were added to the mixed-effects model for serum lipids, inflammatory markers and erythrocyte membrane fatty acid analysis.

## Results and discussion

### Subjects

Six participants withdrew from the study because of family or job pressures or allergies unrelated to treatment foods. Twenty participants completed the study. The average subject age was 38 ± 3 years (range 21-64 years) and BMI was 23 ±1. There were 16 females and 4 males representing several ethnic groups: White (n = 10), Latino (n = 5), Asian (n = 2), and other (n = 3).

### Dietary intake and compliance

The dietary intakes from the 24-hour recalls for energy, carbohydrate, fiber, total fat, saturated fat, and monounsaturated fat were not significantly different between the three treatments (Table 1). As expected, walnut group had higher intake of total polyunsaturated fat (PUFA), total n-6 and n-3 PUFA, LA, ALA compared to the egg treatment groups since walnut treatment group had 1.5 times higher LA and 3 times higher ALA from diet than the egg treatment groups.

**Table 1:** Diet composition from the 24-hour recalls*

**DIET TREATMENTS**							
	**N-3 FA enriched egg**		**Standard egg (control)**		**Walnut**		
**Diet variable**	**LS mean**^**1**^	**Percent energy**	**LS mean**	**Percent energy**	**LS mean**	**Percent energy**	**Global P-value**^**3**^
	**(95% CIs**^**2**^**)**		**(95% CIs**^**2**^**)**		**(95% CIs**^**2**^**)**		
Kcal	1902	--	1895	--	1729	--	0.159
	(1766, 2049)		(1744, 2060)		(1597, 1872)		
KJ	7963	--	7934	--	7239	--	0.159
	(7394, 8579)		(7302, 8625)		(6686, 7838)		
Protein (g)	69^a^	14.51	59^a^	12.45	54^b^	12.49	<0.013
	(62, 76)		(53, 67)		(48, 61)		
Carbohydrate (g)	281	50.10	278	58.68	248	57.37	0.172
	(255, 309)		(250, 309)		(224, 275)		
Total fat (g)	58	27.4	58	27.6	64	33.3	0.537
	(50, 67)		(50, 68)		(55, 75)		
Saturated (g)	16.3	7.71	15.9	7.55	15.1	7.86	0.8138
	(13.7, 19.4)		(13.1, 19.4)		(12.5, 18.1)		
MUFA (g)	21.9	10.4	21.8	10.4	20.1	10.5	0.7558
	(18.4, 26.1)		(17.9, 26.4)		(16.6, 24.2)		
PUFA (g)	13.8^a^	6.53	14.7^a^	6.98	22.5^b^	11.7	<0.0001
	(11.8, 16.1)		(12.3, 17.5)		(19.0, 26.6)		
Total n-6 (g)	11.0^a^	5.21	12.3^a^	5.84	18.9^b^	9.84	<0.0001
	(9.4, 12.9)		(10.3, 14.6)		(16.0, 22.3)		
Linoleic (g)	10.9^a^	5.16	12.2^a^	5.79	18.9^b^	9.84	<0.0001
	(9.3, 12.7)		(10.3, 14.5)		(16.0, 22.3)		
Arachidonic (g)	1.14^a^	0.54	1.05^b^	0.50	1.01^c^	0.53	<0.0001
	(1.09, 1.18)		(1.04, 1.07)		(1.00, 1.02)		
Total n-3 (g)	1.16^a^	0.55	1.38^a^	0.66	3.10^b^	1.61	<0.0001
	(0.93, 1.44)		(1.08, 1.75)		(2.65, 3.63)		
α-Linolenic (g)	1.04^a^	0.49	1.35^a^	0.64	3.07^b^	1.61	<0.0001
	(0.86, 1.26)		(1.09, 1.67)		(2.50, 3.76)		
EPA (g)	0.128^a^	0.06	0.003^b^	0.00	0.001^b^	0.00	<0.0001
	(0, 0.676)		(0, 0.020)		(0, 0.015)		
DHA (g)	0.126^a^	0.06	0.021^b^	0.00	0.002^c^	0.00	<0.0001
	(0, 0.378)		(0, 0.080)		(0, 0.015)		
Cholesterol (mg)	214^a^	--	136^a^	--	46^b^	--	<0.0002
	(131, 351)		(78, 236)		(27, 77)		
Fiber (g)	25	--	28	--	25	--	0.458
	(22, 29)		(24, 32)		(21, 28)		

^*^Means of three 24-hour diet recalls.

^1^Least squares means (LS Means), after back transformation, from 2-way ANOVA with fixed effects for treatment and subject performed on log-transformed data. For EPA and DHA, the recalls for each subject were averaged, and the mean of these subject means is presented. Values in the same row with different superscript letters are significantly different p < 0.05.

^2^Ranges, instead of 95% CIs, are given for EPA and DHA.

^3^P-values respectively for N-3 fatty acid enriched egg and standard egg, N-3 fatty acid enriched egg vs. walnut, and standard egg vs. walnut, adjusted for multiple comparisons by the Tukey-Cramer method; except for EPA and DHA which were adjusted by using the Conover method.

Similarly egg treatment groups had higher protein, arachidonic acid and cholesterol than walnut treatment group. In addition the n-3 FA enriched egg treatment group had higher EPA and DHA than the other two treatment groups and the standard egg treatment had higher DHA than the walnut treatment group which more or less reflects the fatty acid composition of the treatment food. The higher ALA in standard egg group compared to n-3 fatty acid enriched group although not significant could reflect other sources of ALA in the diet and not the treatment per se.

Mixed linear model analyses for changes in subject body weight and body composition were not significant (data not shown), indicating that participants maintained energy balance even after adding a treatment food to their habitual diets. The body composition data established confidence in the 24-hour recall information since calorie intake and weight change was not significantly different during each treatment.

Incorporation of fatty acids into the erythrocyte membrane is presented in Table 2. As expected, LA and ALA were significantly higher during the walnut compared to the egg treatments. DHA was higher during the n-3 fatty acid enriched egg compared to the standard egg and walnut treatment periods. EPA was not significantly different between treatments. There was also a slight decrease in monounsaturated fat in the walnut diet compared to the egg treatments.

**Table 2:** Fatty acid composition of erythrocyte membranes before and after each of the three dietary treatments

**DIET TREATMENTS**							
**Fatty acid**^**1**^	**Standard egg (control)**		**N-3 fatty acid enriched egg**		**Walnut**		**Global *P *** **value**^**3**^
**μg/mole%**	**Baseline**	**End**^**2**^	**Baseline**	**End**^**2**^	**Baseline**	**End**^**2**^	
Saturated	41.1	41.0	40.9	40.9	41.4	41.2	0.9579
	(40.7, 41.5)	(40.2, 41.9)	(40.5, 41.3)	(39.7, 42.1)	(40.8, 42.0)	(40.0, 42.3)	
Trans^4^	0.05	0.02	0 .06	0.03	0 .06	0.03	0.5076
	(0.04, 0.06)	(0.01, 0.03)	(0.04, 0.08)	(0.02, 0.06)	(0.05, 0.07)	(0.02, 0.05)	
Monounsaturated	15.1	15.3	15.4	15.4	14.9	14.4	**<0.0003**
	(14.8, 15.4)	(14.8, 15.7)^a^	(15.2, 15.7)	(15.0, 15.9)^a^	(15.2, 14.6)	(13.9, 14.8)^b^	
Polyunsaturated	36.3	35.6	35.6	35.8	36.1	36.0	0.1761
	(35.9, 36.7)	(34.8, 36.4)	(35.3, 35.9)	(35.0, 36.7)	(34.5, 36.5)	(35.8, 37.4)	
n-6 (total)	30.4	29.7	29.8	29.7	30.0	30.5	0.2880
	(30.1, 30.7)	(29.1, 30.4)	(29.4, 30.2)	(28.7, 30.6)	(29. 6, 30.4)	(29.6, 31.5)	
Linoleic	11.5	10.9	11.3	10.8	11.4	11.9	**<0.0001**
	(11.3, 11.7)	(10.5, 11.3)^a^	(11.0, 11.6)	(10.4, 11.3)^a^	(11.1, 11.7)	(11.5, 12.3)^b^	
Arachidonic	13.6	13.4	13.2	13.6	13.3	13.4	0.7111
	(13.3, 13.9)	(13.0, 13.7)	(12.9, 13.5)	(13.2, 13.9)	(13.02, 13.6)	(13.0, 13.7)	
n-3 (total)	5.7	5.68	5.6	5.97	6.0	5.79	0.0903
	(5.5, 5.9)	(5.48, 5.88)	(5.4, 5.8)	(5.76, 6.17)	(5.76, 6.24)	(5.59, 5.98)	
α-Linolenic	0 .14	0.14	0.13	0.15	0.18	0.19	**<0.0000**
	(0.13, 0.15)	(0.12, 0.16)^a^	(0.12, 0.14)	(0.13, 0.16)^a^	(0.17, 0.19)	(0.18, 0.20)^b^	
EPA^5^	0.28	0.34	0.31	0.30	0.30	0.31	0.1824
	(0.25, 0.31)	(0.31, 0.38)	(0.29, 0.33)	(0.26, 0.33)	(0.27, 0.33)	(0.27, 0.34)	
DHA^5^	2.79	2.90	2.72	3.15	3.12	2.71	**<0.0015**
	(2.64, 2.94)	(2.74, 3.05)^b^	(2.57, 2.87)	(3.00, 3.32)^a^	(2.90, 3.34)	(2.55, 2.87)^b^	

^1^N = 18. Fatty acid values are presented in least square means with 95% confidence intervals. Values within a row with different superscript letters are significantly different, *P* < 0.05.

^2^End values are baseline-adjusted according to the method of Kenward and Roger.

^3^Mixed effects model, controlling for treatment and period effect, adjusted for multiple comparisons using Tukey-Kramer.

^4^Analysis was performed after log-transformation. Results were transformed back into original units and are presented as geometric mean and CI.

^5^EPA = eicosapentaenoic acid; DHA = docosahexaenoic acid.

### Serum cardiovascular disease risk markers

The serum lipid and lipoprotein values following the three treatments are presented in Table 3. On the walnut treatment there was a significant decrease in total cholesterol, triglyceride and apo B, compared to the standard egg but not the n-3 fatty acid enriched egg treatment. However the total-C: HDL-C ratio was lower in the walnut treatment compared to both the egg treatments. There were no significant differences (p > 0.05) among treatments in any of the inflammatory markers assessed in this study (Table 4).

**Table 3:** Serum lipids and lipoproteins before and after each of the three dietary treatments

**DIET TREATMENTS**							
	**Standard egg (control)**		**n-3 FA enriched egg**		**Walnut**		**Global *P *** **Value**^**3**^
**Lipid variable**^**1**^	**Baseline**	**End**^**2**^	**Baseline**	**End**	**Baseline**	**End**	
Cholesterol(s)							
Total (mmol/L)	4.79	5.09	4.62	4.96	4.79	4.77	**<0.0348**
	(4.67, 4.91)	(4.92, 5.26)^a^	(4.50, 4.74)	(4.79, 5.12)^ab^	(4.67, 4.92)	(4.60, 4.94)^b^	
HDL^4^ (mmol/L)	1.29	1.33	1.27	1.30	1.28	1.31	0.7736
	(1.17, 1.41)	(1.25, 1.41)	(1.15, 1.40)	(1.23, 1.38)	(1.15, 1.40)	(1.23, 1.39)	
LDL (mmol/L)	2.75	3.03	2.63	2.96	2.81	2.86	0.2866
	(2.63, 2.87)	(2.87, 3.19)	(2.50, 2.75)	(2.80, 3.12)	(2.69, 2.94)	(2.70, 3.02)	
LDL:HDL	2.13	2.31	2.06	2.32	2.20	2.19	0.1432
	(2.03, 2.25)	(2.19, 2.43)	(1.97, 2.18)	(2.20, 2.44)	(2.00, 2.40)	(2.07, 2.31)	
Total cholesterol:HDL	3.71	3.86	3.63	3.84	3.76	3.64	**<0.0136**
	(3.48, 4.00)	(3.72, 4.00)^a^	(3.40, 3.91)	(3.70, 3.98)^a^	(3.51, 4.05)	(3.50, 3.79)^b^	
Triacylglycerides^4^ (mmol/L)	1.15	1.12	1.13	0.97	1.13	0.92	**<0.0434**
	(1.10, 1.21)	(1.00, 1.26)^a^	(1.07, 1.18)	(0.87, 1.08)^ab^	(1.07, 1.18)	(0.83, 1.03)^b^	
Apolipoprotein A (g/L)	1.54	1.62	1.56	1.59	1.57	1.59	0.6416
	(1.49, 1.59)	(1.57, 1.67)	(1.51, 1.61)	(1.53, 1.64)	(1.53, 1.62)	(1.54, 1.64)	
Apolipoprotein B (g/L)	0.84	0.94	0.82	0.89	0.89	0.86	**<0.0211**
	(0.77, 0.91)	(0.90, 0.98)^a^	(0.77, 0.87)	(0.85, 0.93)^ab^	(0.82, 0.96)	(0.82, 0.90)^b^	
Triacylglycerides: Apo B^4^	1.37	1.10	1.38	1.02	1.27	1.01	0.4541
	(1.33, 1.43)	(0.99, 1.22)	(1.36, 1.39)	(0.91, 1.13)	(1.23, 1.31)	(0.91, 1.12)	
Apo B: Apo A	0.55	0.59	0.52	0.56	0.57	0.54	0.0527
	(0.52, 0.57)	(0.56, 0.62)	(0.51, 0.54)	(0.54, 0.59)	(0.54, 0.59)	(0.51, 0.57)	

^1^Values within a row with different superscript letters are significantly different, P < 0.05. All results are presented as geometric mean and 95% CI.

^2^End values are baseline- adjusted according to the method of Kenward and Roger.

^3^Mixed effects model, controlling for treatment and period effect, adjusted for multiple comparisons using Tukey-Kramer.

^4^Analysis performed after log-transformation. Results have been transformed back into original units and presented as geometric mean and 95% CI.

**Table 4:** Serum inflammatory markers after each of the three dietary treatments

**DIET TREATMENTS**				
**Inflammatory marker**	**Standard egg (control)**	**n-3 FA enriched egg**	**Walnut**	**Global *P*-value**^**1**^
TNF-α^2^ (pg/mL)	0.33 (0.15, 0.72)	0.30 (0.14, 0.65)	0.32 (0.15, 0.69)	0.30
E-selectin^2^ (ng/mL)	0.79 (0.55, 1.12)	0.72 (0.51, 1.02)	0.77 (0.55, 1.10)	0.43
IL-1^2^ (pg/mL)	3.10 (2.52, 3.82)	3.19 (2.60. 3.92)	2.99 (2.43, 3.67)	0.71
IL-6^2^ (pg/mL)	0.89 (0.66, 1.19)	0.97 (0.72, 1.29)	0.79 (0.59, 1.06)	0.23
sICAM^3^ (ng/mL)	9.13 (8.20, 10.06)	9.42 (8.79, 10.05)	9.78 (8.84, 10.73)	0.81
hs-CRP^3^ (ng/mL)	1.95 (1.39, 2.51)	2.64 (1.33, 3.96)	2.36 (1.31, 3.42)	0.75

^1^Global P-value for significant difference among treatments.

^2^P-values were determined by using mixed linear models, adjusting for subject and period effects, on log-transformed data. Results are least square means and 95% CIs after back-transformation.

^3^P-values determined by using Friedman’s chi-squared test.: TNF-α-Tumor necrosis factor-α; IL-Interleukin; sICAM-Soluble intracellular adhesion molecule; hs-CRP-High sensitivity c-reactive protein.

There is substantial evidence that eating fatty fish at least twice a week offers cardiovascular protection [1-3]. However, for vegetarians and for those that do not include fish in their diet for a variety of reasons, other dietary sources of long chain n-3 fatty acids EPA and DHA are n-3 fatty acid enriched eggs and foods fortified or enriched with n-3 fatty acids [1,6]. For vegans it is even more difficult to rely on diet alone to obtain long chain n-3 fatty acids and they may have to resort to supplements made from microalgae oils [9]. Although the body can convert the plant n-3 fatty acid ALA into EPA, the conversion to DHA seems to be less efficient [10]. In this study we compared ALA rich walnuts and n-3 fatty acid enriched (primarily DHA) eggs with a standard egg control with respect to their effects on select CVD risk factors among lacto-ovo-vegetarians (LOV). Adding ~24 g/d (1 ounce, 6 d/week) of walnuts to the habitual diet of LOV lowered total-C and TAG compared to eating 6 standard eggs a week. More importantly, adding ALA rich walnuts to the habitual diet of LOV lowered the ratio of total: HDL cholesterol compared to both egg treatments, even when the egg contained a significant amount of DHA.

Previous studies have shown that feeding 42-57 grams of walnuts per day (1.5-2 ounces/d) lowers total-C, LDL-C and TAG in both normal and hypercholesterolemic individuals [21,22,25-28]. While we showed a significant lowering in total-C and TAG, LDL-C remained unchanged. This could either be due to lower baseline LDL-C levels in our normocholesterolemic subjects or because of the low dose of walnuts (‵24 g/d) used in our study compared to higher amounts typically used in other feeding studies [14,21,22,25-27]. The lowering of serum total cholesterol by walnuts is consistent with previous findings (13, 20). While it seems that this is due to a lower intake of cholesterol in the walnut treatment compared to the egg treatments, it is known that dietary fatty acids are better predictors of serum cholesterol than dietary cholesterol per se. In fact we have observed decreases in serum cholesterol following walnut enriched diet even when dietary cholesterol intake was similar to the control diet (20).

In previous studies it has been observed that DHA increases LDL-C [14,29]. It was interesting however; that the n-3 fatty acid enriched egg treatment resulted in cholesterol levels lower than the standard egg treatment although the values were not significant statistically. This may be attributed to the higher ALA content (1 g/egg yolk) of the n-3 fatty acid enriched eggs (almost half of what was found in the one ounce of walnuts) which were derived from flaxseed meal fed chicken compared to only 0.1 g ALA per standard egg yolk.

In a previous study comparing fatty fish and walnuts, we showed that walnuts lowered total and LDL-C, while the fatty fish lowered TAG and increased HDL-C compared to the other treatment [14]. In the present study, TAG decreased 13% (not statistically significant) on the n-3 fatty acid enriched egg treatment and 18% on the walnut treatment compared to the standard egg treatment. 13-18% decrease is clinically relevant and suggests that ALA and EPA/DHA can both lower triglyceride levels. In our previous study with fatty fish and walnuts we showed a two fold increase in erythrocyte EPA levels following fatty fish intake compared to walnuts and a corresponding lowering of TAG only in the fatty fish diet group [14]. In this study the EPA in erythrocyte membrane were not different among treatments but DHA was significantly higher in the n-3 fatty acid enriched egg treatment compared to the other two treatments. These observations do not help explain the greater TAG lowering seen with walnut treatment. However, the total n-3 fatty acids derived from the diet was 3 times higher in the walnut treatment compared to the egg treatments. Perhaps this may explain why walnuts produced a more significant effect on TAG than the n-3 fatty acid enriched eggs.

The total: HDL cholesterol ratio is considered a clinical benchmark to assess risk of CVD [30]. Walnut treatment was most effective in reducing this ratio compared to both the egg treatments. Along with this ratio, the Apo B: Apo A-1 ratio also approached significant difference between treatments with a lower ratio after the walnut treatment compared to the standard egg treatment. This suggests that overall a more favorable lipid profile was achieved through eating 1 ounce of walnuts 6 times a week compared to eating 6 standard eggs.

These effects of walnuts may be mediated partly by the fatty acid composition of walnuts but may extend beyond the lipid matrix of the walnut. The EPA levels in erythrocyte membrane are similar in all three treatments. Although EPA levels in the walnut treatment group did not increase as expected, this might be due to the high n-6 fatty acid intake. On the other hand the DHA level in erythrocyte membrane in the walnut treatment was significantly lower than the n-3 FA enriched egg treatment. This suggests that consumption of ALA rich foods alone may not be sufficient to achieve DHA status in LOVs when compared to LOVs that consume a direct source of DHA. Future research looking at a higher dose of walnuts or other ALA rich foods, or a diet treatment longer than 8 weeks may be required to determine if erythrocyte incorporation of DHA increases under these conditions. However for the cardiovascular risk markers measured, the levels of DHA in erythrocyte membrane does not seems to be a limiting factor, at least in this study.

Inflammation plays a role in the development and progression of atherosclerosis and contributes to an increased risk of CVD [7]. There is some evidence to suggest that n-3 fatty acids may lower inflammation [7,8,22], as indicated by serum markers of inflammation, but these finding are not consistent [31,32]. In our study we did not show any significant changes in inflammatory markers following either walnut or n-3 fatty acid enriched egg treatments. Most of the studies that have looked at healthy individuals and found no effect of n-3 fatty acids on inflammatory biomarkers especially the cytokines [31]. However, the soluble adhesion molecules sICAM-1 and sVCAM-1 were decreased following intake of n-3 fatty acids in healthy subjects but only at high dose (~2 g/d of EPA and DHA) or with longer duration of intervention (12 weeks) [32]. Subjects in our study on the n-3 fatty acid enriched eggs obtained ~500 mg DHA per egg yolk but very little EPA (~30 mg/egg yolk) and for only 8 weeks. A similar beneficial effect of walnuts on the adhesion molecule sE-selectin has been observed, but only when walnuts and other ALA rich foods contributed at least 6.5-12% of the total energy of the diet [33-35]. Reducing levels of cellular adhesion molecules will have a significant role in modulating the atherosclerotic process and therefore, future studies considering dietary modification with n-3 fatty acids must be mindful of appropriate dose and duration especially, among healthy individuals.

Previously we observed that plasma levels of eicosanoids such as prostaglandin (PGE_2_) and thromboxane (TXA_2_) metabolites were lowered following the intake of two fatty fish meals (EPA + DHA of 780 mg/d) per week [34]. It is known that the eicosanoids produced from EPA is anti-inflammatory and those derived from arachidonic acid pro-inflammatory [36]. The effect that we observed was also seen with walnut intake (42.5 g/d) in that same study, which is attributed to not just the ALA content but also to the γ-tocopherol content of walnuts, known to inhibit the activity of cyclooxygenase enzyme [37]. Since we did not measure these biomarkers in the present study, whether or not these markers would have been influenced following the consumption of low dose walnuts or n-3 fatty acid enriched eggs remains unresolved.

This was a free-living study with the rigor of control not nearly the same as in metabolically controlled studies. On the other hand, free living conditions in which subjects follow their habitual diet with very little dietary modification provides more generalizable results since it reflects more realistically what the general population would eat. Since this study was done in healthy LOVs who limit the amount of animal products in their diet, caution must be used in interpretation of the results for people with chronic diseases, people that consume other animal products and people that may exhibit sensitivity to dietary cholesterol.

## Conclusion

Our study suggests that for lacto-ovo-vegetarians, replacing standard eggs with n-3 fatty acid enriched eggs in their diet may be an option to increase DHA content of erythrocyte membranes. However, to influence blood lipid risk factors, it seems that eating a handful of walnuts daily may have overall better benefits for lacto-ovo-vegetarians.

## Abbreviations

ALA: α-linolenic acid; CVD: Cardiovascular disease; DHA: Docosahexaenoic acid; EPA: Eicosapentaenoic; FA: Fatty acids; HDL-C: High density lipoprotein cholesterol; Hs-CRP: High- sensitivity C-reactive protein; IL-1β: Interleukin 1β; IL-6: Interleukin 6; LOV: Lacto-ovo-vegetarians; LDL-C: Low density lipoprotein cholesterol; FA: n-3 Fatty acids n-3; PGE2: Prostaglandin 2; RD: Registered dietitian; sICAM-1: Soluble intracellular adhesion molecule-1; Total-C: Total cholesterol; TXA2: Thromboxane 2; TAG: Triacylglycerol; TNF-α: Tumor necrosis factor alpha.

## Competing interests

There are no conflicts of interest. This work was supported by the American Egg Board Fellowship; Agriculture Research Institute (Grant) from California State Polytechnic University, Pomona; California Walnut Commission (in-kind donation of walnuts); Chino Valley Ranchers (in-kind donation of eggs). Several authors (JS, EH, SR) have received previous research grants from the walnut and other nut industries. BBW received a dissertation fellowship grant from the American Egg Board, and a grant from the Agriculture Research Institute, both of which were used entirely to fund this study. BBW received a prior grant from the disbanded California Egg Commission to compile recent egg-related research in 1999.

## Authors’ contributions

BBW was responsible for the study conception and design, subject recruitment and selection, interpretation of the data, management of the feeding trial, drafting of the manuscript, and obtaining the funding for the study. EH provided lab support. SR helped with study design, performed subject recruitment and selection, interpreted the data, reviewed and edited the manuscript. EH and JS helped develop the study design, assisted in data interpretation and reviewed the manuscript. All authors read and approved the final manuscript.
